# Pemafibrate Pretreatment Attenuates Apoptosis and Autophagy during Hepatic Ischemia-Reperfusion Injury by Modulating JAK2/STAT3*β*/PPAR*α* Pathway

**DOI:** 10.1155/2021/6632137

**Published:** 2021-03-11

**Authors:** Ziqi Cheng, Chuanyong Guo

**Affiliations:** Department of Gastroenterology, Shanghai Tenth People's Hospital, Tongji University School of Medicine, Shanghai 200072, China

## Abstract

Hepatic ischemia-reperfusion injury (HIRI) is a common phenomenon in liver transplantation and liver surgery. This article is aimed at clarifying the role of pemafibrate in HIRI through JAK2/STAT3*β*/PPAR*α*. In the experiment, we divided Balb/c into seven groups, namely, normal control (NC), Sham, PEM (1.0 mg/kg), IRI, IRI + PEM (0.1 mg/kg), IRI + PEM (0.5 mg/kg), and IRI + PEM (1.0 mg/kg). We used biochemical assay, histopathological evaluation, immunohistochemistry, RT-PCR and qRT-PCR, ELISA analysis, and other methods to determine the level of serum AST, ALT, IL-1*β*, and TNF-*α* in the liver at three time points (2 h, 8 h, and 24 h) after reperfusion of apoptosis factor, autophagy factor, and the JAK2/STAT3/PPAR*α* content in tissues. Our experiment results showed that the pemafibrate can effectively reduce the level of hepatic IR injury. In addition, pemafibrate has anti-inflammatory, antiapoptotic, and antiautophagy effects, which are mediated by the JAK2/STAT3*β*/PPAR*α* pathway.

## 1. Introduction

Hepatic ischemia-reperfusion injury (HIRI) is the injury caused by reperfusion after liver ischemia [1, 2]. After the blood supply to liver tissue was interrupted due to liver ischemia, the subsequent blood reperfusion brings in a large number of inflammatory cells, which leads to serious damages to the structure and function of the liver [3–5]. Ischemia-reperfusion injury is a complicated pathophysiological process. A large number of studies have shown that HIRI, involving amounts of cells and multiple molecular mechanisms, is characterized by oxidative stress and the release of reactive oxygen species (ROS) [3, 6–8]. ROS is the starting point that causes a cascade of reactions dominated by inflammatory cells, cytokine release, apoptosis, and autophagy [9, 10]. This damage not only affects the liver but also has serious negative effects on the brain, heart, kidneys, and gastrointestinal tract, which is a complex systemic process.

When senescent macromolecular substances appear in and damage the cells, the cells will initiate programmed cell death. Autophagy, a kind of programmed cell death, is a complex process that ensures the normal function of cells and recirculates the digested materials to efficiently maintain the normal activities of cells [11]. Although autophagy is considered to be a self-protection mechanism, excessive upregulation can also cause cell death [12]. Apoptosis, another kind of programmed cell death, has very obvious morphological characteristics, including cell volume reduction, nuclear chromatin shrinkage, and the formation of apoptotic bodies. There are two core molecular families of the apoptosis pathway: the BCL-2 family and the Caspase family [13].

Janus kinase (JAK)/signal transducer and activator of transcription (STAT) participate in signal transduction induced by a number of cytokines and interferons in vivo. After receiving the signal from JAK2, STAT3 forms the dimer after phosphorylation and enters the nucleus to regulate the transcription of related genes. STAT3 is mainly involved in regulating cell proliferation, differentiation, apoptosis, and inflammation [14–16]. There are two subtypes of STAT3: STAT3*α* and STAT3*β*. Interestingly, studies have shown that STAT3*α* and STAT3*β* have opposite effects on cancer, but the role of STAT3*β* in cells is still controversial [17].

Peroxisome proliferator-activated receptor (PPAR) is a member of the nuclear receptor superfamily [18]. As a transcription factor, PPAR establishes a link between transcription and signal molecules, including PPAR*α*, PPAR*β*, and PPAR*γ*. PPAR*α* is highly expressed in the liver, intestinal epithelial cells, and cardiac muscle cells and plays an important role in fatty acid oxidation. It will combine with retinoid X receptor (RXR) to form a heterodimer, activate the PPAR Response Element (PPRE) located in the upstream of the target gene, and participate in the regulation of nuclear factor *κ*B (NF-*κ*B) and activator protein 1 (AP-1) [18, 19]. Pemafibrate, a new type of selective PPAR*α* modulator (SPPARM *α*), enhances PPAR*α*'s activity and has high selectivity by introducing side chains into fibric acid. These side chains later form a Y-shaped structure and fill in the ligand binding site of PPAR*α*, thereby promoting massive activation of PPAR*α*. Compared with other fibrates, pemafibrate has a feature of basic EC50 value and higher selectivity. Pemafibrate could better aim at specific targets and reduce the risk of binding multiple sites.

Although there are many studies on HIRI, neither the molecular mechanism nor effective drugs to this injury was still not identified. Therefore, to find out the specific drugs that can alleviate HIRI and to clarify the drug action mechanism are urgent problems to be solved at present. We hypothesized that pemafibrate can alleviate HIRI by inhibiting inflammation, apoptosis, and autophagy and conducted the following experiments.

## 2. Materials and Methods

### 2.1. Reagents

The pemafibrate was purchased from MedChemExpress (Monmouth Junction, NJ, USA) and used by adding 10% DMSO and 90% corn oil in our experiments. The microplate test kits used for measuring the levels of alanine aminotransferase (ALT) and aspartate aminotransferase (AST) were bought from the Jiancheng Bioengineering Institute (Jiancheng Biotech, Nanjing, China). Enzyme-linked immunosorbent assay (ELISA) kits were acquired from Anogen (Ontario, Canada). The PrimeScript RT Reagent Kit and SYBR Premix Ex Taq were purchased from TaKaRa Biotechnology (Dalian, China).

During the whole experimental process, we used many antibodies, including anti-Bax, anti-caspase 3, anti-caspase 9, anti-Beclin-1, PPAR*α* (Proteintech, Chicago, IL, USA), anti-TNF-*α*, anti-Bcl-2, anti-microtubule associated protein 1 light chain 3 (LC3), anti-JAK2, anti-STAT3, anti-p-STAT3 (Cell Signaling Technology, Danvers, MA, USA), anti-IL-6, and anti-IL-1*β* (Antibody Revolution, San Diego, CA, USA).

### 2.2. Animal Preparation

The protocol of this study was approved by the Animal Care and Use Committee of Shanghai Tongji University. We handled and took care of the animals under the guidance of the National Institutes of Health Guidelines and tried our best to minimize the pain and suffering of mice throughout the whole experiment. We raised the male Balb/c mice (6–8 weeks old, 23 ± 2 g) purchased from the Shanghai SLAC Laboratory Animal Co., Ltd. (Shanghai, China) in a clean and temperature-controlled environment at 24°C ± 2°C under a 12 h : 12 h/light : dark cycle. In the environment, standard laboratory food and water were available freely for mice.

### 2.3. Experimental Design

We divided the one hundred and two mice randomly into seven groups as follows:

1. *Normal Control (NC)*. Six mice were only injected with vehicle (10% DMSO and 90% corn oil)
2. *Sham*. Eighteen mice took laparotomy after anesthesia, and their abdominal cavity was stitched without IRI
3. *PEM (1.0 mg/kg)*. Six mice were only injected with 1.0 mg/kg PEM
4. *IRI*. Eighteen mice suffered ischemia and reperfusion
5. *IRI + PEM (0.1 mg/kg)*. Before IRI, eighteen mice were injected with 0.1 mg/kg PEM for 5 days
6. *IRI + PEM (0.5 mg/kg)*. Before IRI, eighteen mice were injected with 0.5 mg/kg PEM for 5 days
7. *IRI + PEM (1.0 mg/kg)*. Before IRI, eighteen mice were injected with 1.0 mg/kg PEM for 5 days [20–22]

Given the pharmacokinetics and initial experiment of PEM for 5 days before IRI, a certain dose of PEM determined by a previous study and initial experiments was injected into mice's abdomens. At 2 h, 8 h, or 24 h after IRI, we randomly killed six mice in each group and then collected blood and liver tissues for further experiments.

### 2.4. Establishment of a Hepatic IRI Mouse Model

In this experiment, we established a warm hepatic IRI animal model. Before surgery, we kept mice not taking food for twelve hours but allowed them to drink water freely, then used sodium pentobarbital (40 mg/kg, 1.25%) (Nembutal, St Louis, MO, USA) to anesthetize the mice by intraperitoneal injection. When the algesia of these mice disappears completely, we started to perform laparotomy. After disinfecting the skin with alcohol, we made an incision along the linea alba and entered into the abdominal cavity through the incision. After spotting the liver, we turned the hepatic lobes over to expose the first porta hepatis. Following that, we clipped the portal vein, hepatic artery, and common bile duct with microarterial clamps to block the hepatic blood flow. Once hepatic ischemia occurred, the color of liver lobes turned immediately from dark red to pale red. Then, we placed the mice on an electric blanket to maintain their body temperature with a humid saline gauze covered on their incisions. We removed the clamps after blocking the blood flow for 45 min and then let the blood flow back to the liver, by which the liver completed a reperfusion process. In the final step, we stitched the abdominal cavity and placed these mice on electric blankets [23, 24].

### 2.5. Biochemical Assays

We collected the orbital blood samples of the mice who had suffered hepatic ischemia and reperfusion processes, then extracted serum from the samples by centrifuging at 2,000 × g at 4°C for 10 min and stored it at −80°C. Following the instructions of the manufacturer protocols, we used the microplate test kits (Olympus AU1000, Olympus, Tokyo, Japan) to detect serum levels of ALT and AST and used the ELISA kits to measure serum levels of IL-1*β* and TNF-*α*.

### 2.6. Histopathological Evaluation

We removed the liver from the abdomen cavity of mice, then put the liver tissue in 4% paraformaldehyde for twenty-four hours for renovation. On the next day, we dehydrated it with ethanol of different concentrations. After that, we embedded paraffin into the tissues and cut them into slices about 3 *μ*m thick. Finally, we stained the slices with hematoxylin and eosin (H&E) to observe the degree of damage.

### 2.7. Immunohistochemistry

We put these liver slices in a baking oven at 60°C for 20 min to remove the residual wax on them and then rehydrated the slices with xylene and ethanol. After that, we retrieved the antigen by heating the slices at 95°C for 10 min and then cooled them to 25°C. Blocking endogenous peroxidase activity was achieved by immersing the slices in hydrogen peroxide (H_2_O_2_) solution (3%) for 20 min at 37°C. In order to avoid generating high backgrounds, we used 5% bovine serum albumin to block nonspecific binding sites for 1 h. Then, the liver slices were incubated overnight with primary antibodies against Bcl-2 (1 : 500), Bax (1 : 500), Beclin-1 (1 : 500), and LC3 (1 : 500) at 4°C. In the next morning, we added the slices into a secondary antibody which can bond the primary antibodies specifically and incubated them for 1 h at 37°C. The efficacy of antibody binding can be detected by a diaminobenzidine kit. Lastly, we observed slices under an optical microscope.

### 2.8. Reverse Transcription Polymerase Chain Reaction (RT-PCR) and Quantitative Real-Time PCR (qRT-PCR)

All RNAs of liver tissues were extracted by TRIzol reagent (Thermo Fisher Scientific, Waltham, MA, USA) and then reversely transcribed into cDNA. We performed qRT-PCR by SYBR Premix EX Taq under the guidance of the manufacturer instructions to detect the level of mRNA with a 7900HT Fast PCR System (Applied Biosystems, Foster City, CA, USA). The primers were *β*-actin, forward GGCTGTATTCCCCTCCATCG, reverse CCAGTTGGTAACAATGCCATGT; IL-1*β*, forward GAATGCCACCTTTTGACAGTG, reverse TGGATGCTCTCATCAGGACAG; IL-6, forward CTGCAAGAGACTTCCATCCAG, reverse AGTGGTATAGACAGGTCTGTTGG; TNF-*α*, forward CAGGCGGTGCCTATGTCTC, reverse CGATCACCCCGAAGTTCAGTAG; Bcl-2, forward GCTACCGTCGTCGTGACTTCGC, reverse CCCCACCGAACTCAAAGAAGG; Bax, forward AGACAGGGGCCTTTTTGCTAC, reverse AATTCGCCGGAGACACTCG; Caspase 3, forward CTCGCTCTGGTACGGATGTG, reverse TCCCATAAATGACCCCTTCATCA; Caspase 9, forward GGCTGTTAAACCCCTAGACCA, reverse TGACGGGTCCAGCTTCACTA; Beclin-1, forward ATGGAGGGGTCTAAGGCGTC, reverse TGGGCTGTGGTAAGTAATGGA; LC, forward GACCGCTGTAAGGAGGTGC, reverse AGAAGCCGAAGGTTTCTTGGG; and P62, forward GAGGCACCCCGAAACATGG, reverse ACTTATAGCGAGTTCCCACCA.

### 2.9. Western Blot Analysis

We extracted the protein from liver tissue by homogenizing with radioimmunoprecipitation assay lysis buffer (Kaiji Biology, Nanjing, China), phenylmethane-sulfonyl fluoride, and protease inhibitors. The protein concentration was measured by a bicinchoninic acid assay (Kaiji Biology). Before performing electrophoresis, we boiled the protein samples at 100°C for 5 min, then used 10% or 12.5% SDS-PAGE to separate proteins from protein samples during electrophoresis at 120 V. Next, we chose the wet transfer method to transfer proteins onto polyvinylidene fluoride membranes (Hybond™; Escondido, CA, USA). One hour later, we used 5% nonfat milk to block the nonspecific binding sites for at least 1 hour, then incubated the membranes overnight at 4°C with the following primary antibodies: anti-*β*-actin (1 : 1,000), anti-IL-6 (1 : 500), anti-TNF-*α* (1 : 500), anti-Bcl-2 (1 : 1,000), anti-Bax (1 : 1,000), anti-caspase 3 (1 : 500), anti-caspase 9 (1 : 500), anti-Beclin-1 (1 : 1,000), anti-LC3 (1 : 1,000), anti-JAK2 (1 : 1000), anti-STAT3 (1 : 1000), and anti-p-STAT3 (1 : 1000). In the next morning, we used the 0.1% Tween-contained PBST to wash the membranes for three times, then incubated the membranes with the secondary antibody for 1 h at 37°C, and washed the membranes with PBST for another three times. The aforementioned secondary antibody could be anti-mouse or anti-rabbit antibodies (1 : 2000). Finally, we used an Odyssey two-color infrared laser imaging system (Licor, Lincoln, NE, USA) to observe the chromogenic results.

### 2.10. Statistical Analysis

In order to ensure the veracity of our study, we performed all experiments at least three times and presented the data as mean ± SD. We analyzed the data and results of the serum levels of ALT, AST, Western blot, ELISA, and qRT-PCR were analyzed by Student's *t*-test or two-way analysis of variance (followed by post hoc Dunnett's test). When the *P* values are less than 0.05, we consider the results as statistically significant. All statistical graphs were drawn by GraphPad Prism 8 (GraphPad Software, Inc., San Diego, CA, USA).

## 3. Results

### 3.1. Pemafibrate and Surgery Will Not Produce Side Effects on the Structure and Function of the Liver

**Figure 1:**
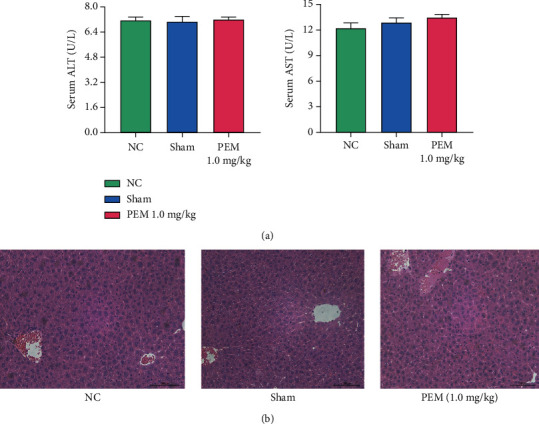
Pemafibrate has no toxic and side effects on liver structure and function. (a) The levels of ALT and AST were shown as mean ± SD by GraphPad Prism 8 (*n* = 6; *P* < 0.01). In these three groups, there was no significant difference in transaminase expression between two groups. (b) After H&E staining, the liver was observed under the light microscope (original magnetization, ×200).

In order to determine whether liver damage is only caused by the model and whether pemafibrate is hepatotoxic, we detected ALT and AST in the blood samples of the NC group, Sham group, and pemafibrate (1.0 mg/kg) group of mice. The results showed that there was no significant statistical difference in the levels of AST and ALT (Figure 1(a)). Observation on the mouse liver tissues with H&E staining found that the structure and function of the liver tissues were not remarkably changed in H&E-stained sections (Figure 1(b)). Therefore, we believe that the concentration of pemafibrate and surgery do not affect liver function.

### 3.2. Pemafibrate Pretreatment Can Relieve Liver IRI

**Figure 2:**
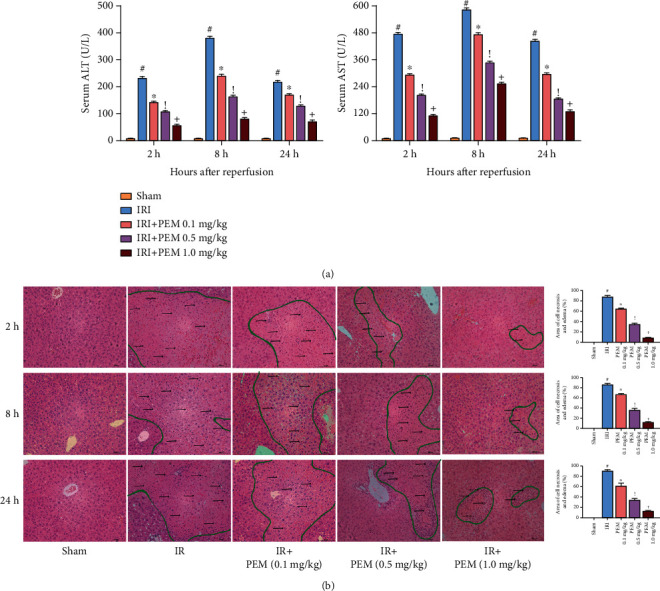
Pemafibrate can significantly alleviate the changes of liver structure and function after ischemia-reperfusion. (a) In three time points, the transaminase level of IRI mice increased significantly and improved after treatment, showing a dose-dependent. ALT and AST levels were expressed by mean ± SD. (b) H&E-stained hepatic sections were examined under light microscopy and imaged at a ×20 magnification. In the IRI group, there were a lot of eosinophilic and unorganized necrotic areas (circled by green lines) and increased inflammatory reactions (indicated by black arrows). However, this phenomenon was alleviated by PEM. Data was given as mean ± SD (*n* = 6, ^#^*P* < 0.05 for Sham versus IRI, ^∗^*P* < 0.05 for IRI + PEM 0.1 mg/kg versus IRI, ^!^*P* < 0.05 for IRI + PEM 0.1 mg/kg versus IRI + PEM 0.5 mg/kg, and ^+^*P* < 0.05 for IRI + PEM 0.5 mg/kg versus IRI + PEM 1.0 mg/kg; (a) two-way ANOVA; (b) Student's *t*-test).

By comparing H&E-stained sections, ALT and AST levels in Sham, IR, low-dose, medium-dose, and high-dose groups, we found that in the three time points, the liver tissues of the IRI group were significantly damaged. However, the level of transaminase of the pemafibrate pretreated group decreased, and the effect of high concentration of pemafibrate in alleviating damage was better than that of low concentration, which was dose-dependent (Figure 2(a)). Observation by optical microscope found that there was no obvious damage in the Sham group, but the liver tissue in the IRI group showed structural changes, such as ballooning, necrosis, and inflammatory cell infiltration. Especially at the time point of 8 h, the liver injury in the IRI group was more severe. However, these changes were relieved in the PEM-treated group. In short, these experimental results once again corroborate the aforementioned conclusion: liver damage is mainly caused by the vascular ligation instead of the surgical operation. In addition, H&E results indicate that pemafibrate can alleviate the inflammatory damage and necrosis of IRI (Figure 2(b)).

### 3.3. Pemafibrate Inhibits Inflammation

**Figure 3:**
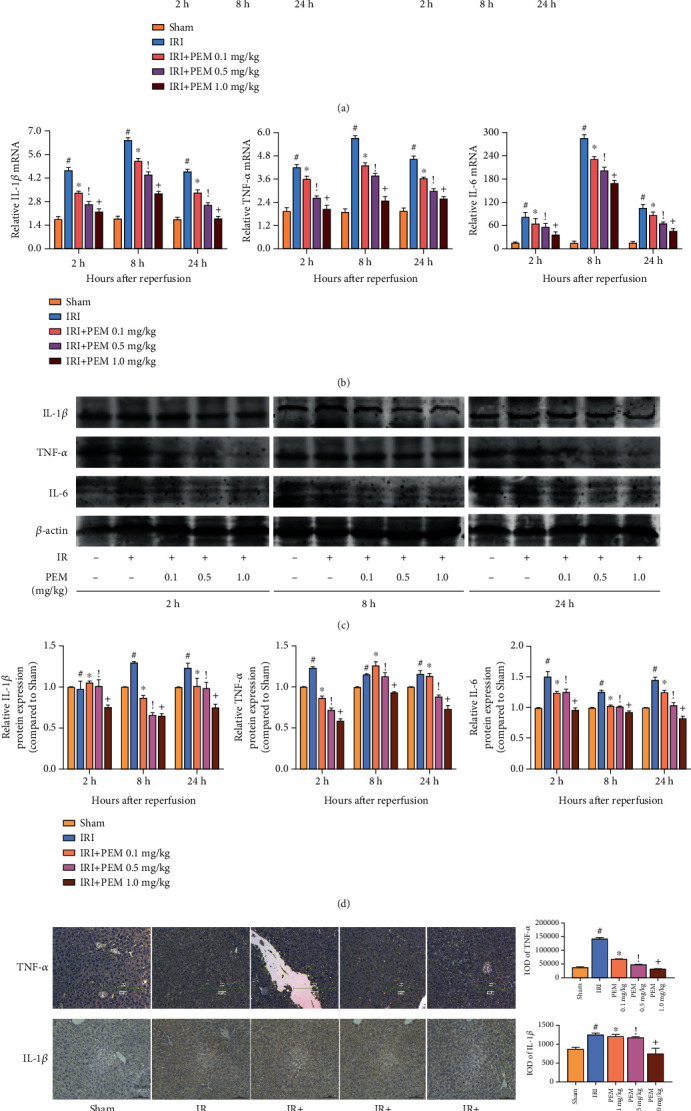
PEM pretreatment inhibits the production of IL-1*β*, IL-6, and TNF-*α* in hepatic IRI. (a) The serum IL-1*β* and TNF-*α* levels were measured by ELISA and given as mean ± SD at 2, 8, and 24 hours after reperfusion in mice. (b) The relative mRNA levels of IL-1*β*, IL-6, and TNF-*α* were evaluated in each group, as shown by qRT-PCR. (c, d) Protein expression of IL-1*β*, IL-6, and TNF-*α* was detected by Western blot. (e) Immunohistochemistry was used to detect TNF-*α* and IL-1*β* expression in liver tissues (original magnification, ×200). The IOD sum was analyzed with the Image-Pro Plus software 6.0. Data was presented as the mean ± SD (*n* = 6, ^#^*P* < 0.05 for Sham versus IRI, ^∗^*P* < 0.05 for IRI + PEM 0.1 mg/kg versus IRI, ^!^*P* < 0.05 for IRI + PEM 0.1 mg/kg versus IRI + PEM 0.5 mg/kg, and ^+^*P* < 0.05 for IRI + PEM 0.5 mg/kg versus IRI + PEM 1.0 mg/kg; (a, b, d) two-way ANOVA; (e) Student's *t*-test). Expression levels of IL-1*β*, TNF-*α*, and IL-6 were significantly increased in IRI mice. PEM pretreatment dramatically reduced the levels, particularly at 1.0 mg/kg.

Based on the above H&E staining results, the expression levels of common inflammatory factors—IL-1*β*, IL-6, and TNF-*α*—were detected. We tested the expression levels in liver tissues by ELISA (Figure 3(a)) and found that in the three time periods, the expression level of IRI group was significantly higher than that of the Sham group. However, under the intervention of pemafibrate, the level of inflammatory factors decreased in a dose-dependent manner. The qRT-PCR (Figure 3(b)) and Western blot analysis (Figures 3(c) and 3(d)) results also showed that IL-1*β* and TNF-*α* were significantly increased. And IHC showed that the levels of IL-1*β* and TNF-*α* were lower than those of the IR group, showing a downward trend (Figure 3(e)). These results indicated that pemafibrate can inhibit the release of inflammatory factors such as IL-1*β*, IL-6, and TNF-*α*.

### 3.4. Pemafibrate Inhibits Programmed Cell Death in HIRI, including Apoptosis and Autophagy

**Figure 4:**
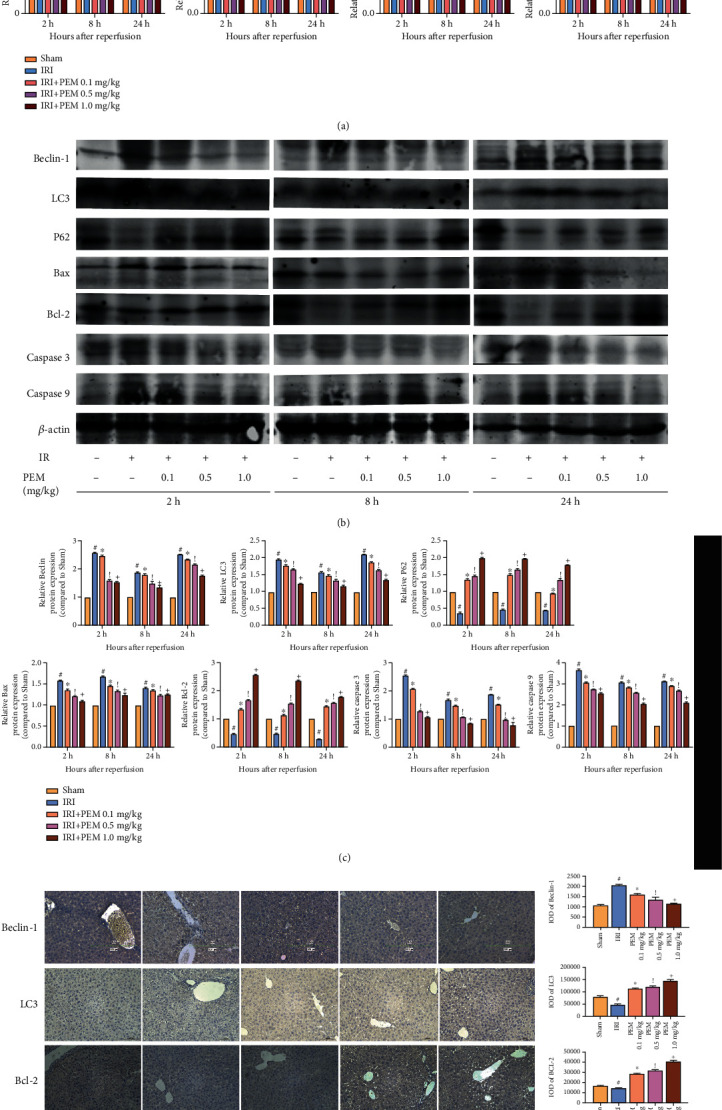
PEM pretreatment ameliorates apoptosis and autophagy in hepatic IRI. (a) The relative mRNA levels of Bcl-2, Bax, Caspase 3, Caspase 9, Beclin-1, LC3, and P62. (b, c) Protein expression of apoptosis- and autophagy-related proteins. (d) Immunohistochemistry was used to detect Bcl-2, Bax, Beclin-1, and LC3 expression in liver tissues (original magnification, ×200). The IOD sum of brown area to total area was analyzed with the Image-Pro Plus software 6.0. Data was presented as the mean ± SD (*n* = 6, ^#^*P* < 0.05 for Sham versus IRI, ^∗^*P* < 0.05 for IRI + PEM 0.1 mg/kg versus IRI, ^!^*P* < 0.05 for IRI + PEM 0.1 mg/kg versus IRI + PEM 0.5 mg/kg, and ^+^*P* < 0.05 for IRI + PEM 0.5 mg/kg versus IRI + PEM 1.0 mg/kg; (a, c) two-way ANOVA; (d) Student's *t*-test). In the IRI group, the expression of P62 and Bcl-2 was significantly lower than that in the NC group, while the others were significantly higher. PEM could reverse this change.

There are extensive programmed cell deaths in HIRI, including in ways of apoptosis and autophagy. The experimental results of extracting mRNA for qRT-PCR and observing the distribution of molecules in the sliced liver tissue were found to be consistent with that of WB (Figure 4(a)). We selected BCL-2, Bax, Caspase 3, and Caspase 9 to detect the degree of apoptosis in HIRI. Western blot result showed that, compared with the level of IR group, the degree of Bcl-2 had a rising trend, and degrees of Bax, Caspase 3, and Caspase 9 decreased significantly (Figures 4(b) and 4(c)). Another form of cell death in HIRI is autophagy. With the use of common detection molecules for autophagy including Beclin-1, LC3 and P62, and Western blot to observe the expression trend of each molecule under different intervention conditions, we found that the expression of P62 at 1.0 mg/kg was higher than 0.01 mg/kg, and Beclin-1 and LC3 showed a downward trend. The IHC results are the same as the previous two (Figure 4(d)). In conclusion, pemafibrate can relieve apoptosis and autophagy in liver ischemia-reperfusion injury.

### 3.5. Pemafibrate Protects the Liver by Regulating the JAK2/STAT3*β*/PPAR*α* Signaling Pathway

**Figure 5:**
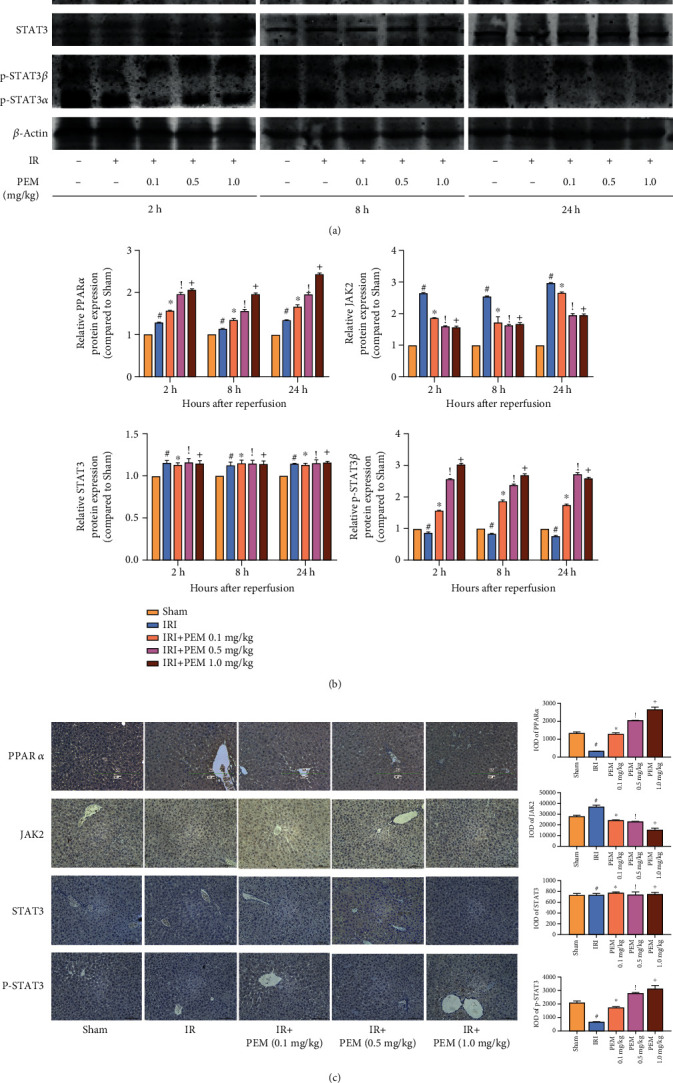
PEM modulates the phosphorylation of JAK2/STAT3*β* in hepatic IRI. (a, b) Protein expression of PPAR*α*, JAK2, STAT3, and p-STAT3. (c) The quantitative analysis of Western blot results of JAK2/STAT3*β*/PPAR*α*. Data was presented as the mean ± SD (*n* = 6, ^#^*P* < 0.05 for Sham versus IRI, ^∗^*P* < 0.05 for IRI + PEM 0.1 mg/kg versus IRI, ^!^*P* < 0.05 for IRI + PEM 0.1 mg/kg versus IRI + PEM 0.5 mg/kg, and ^+^*P* < 0.05 for IRI + PEM 0.5 mg/kg versus IRI + PEM 1.0 mg/kg; (b) two-way ANOVA; (c) Student's *t*-test). The expression of PPAR*α* and p-STAT3*β* was notably increased, but JAK2 saw a steep drop.

We tested JAK2, Stat3, and p-Stat3 and found that the expression level of JAK2 in the PEM-treated group was lower than that in the IRI group, and the levels of Stat3*α* and Stat3*β* did not change significantly. The expression saw a different trend of p-Stat3*α* and p-Stat3*β*, increasing considerably at level of p-Stat3*α* but falling markedly at level of p-Stat3*β*, compared with the IRI group. When pemafibrate acts as a PPAR*α* agonist, Western blot results indicate that under the action of different concentrations of pemafibrate, the expression of PPAR*α* in liver tissues increased sequentially (Figures 5(a) and 5(b)). These results, further verified in immunohistochemistry experiments (Figure 5(c)), indicate that pemafibrate can inhibit the JAK2/STAT3*β*/PPAR*α* signaling pathway.

## 4. Discussion

We measured the serum levels of ALT and AST in 1.0 mg/kg PEM group, normal control group, and Sham group and compared the changes of liver tissue structure after H&E staining, in order to ensure that the drug dose did not damage the liver. We showed that 1.0 mg/kg PEM had no obvious toxic effect on liver. After verifying that the drug has no toxic effect on the liver, we established the hepatic IRI mouse model. The results showed that the levels of both in the IRI group were significantly increased, but pemafibrate could inhibit this change. At the same time, liver necrosis induced by HIRI was observed under light microscope, and the accumulation of inflammatory cells was improved after PEM treatment.

There are many mechanisms for the occurrence and development of HIRI, such as ATP depletion, endothelin (ET)/nitric oxide ratio imbalance, Ca_2_^+^ overload, and macrophage activation [25]. In this process, a large amount of ROS is released, which stimulates the cascade of immune cells. Immune cells are composed of Kupffer cells, natural killer cells, and dendritic cells. Activated macrophages secrete a large number of inflammatory factors, such as IL-1*β*, IL-6, and TNF*α* [4, 26, 27]. Based on previous studies, we detected the levels of IL-1*β*, IL-6, and TNF-*α* in liver tissues and observed the liver tissues under a microscope. A number of inflammatory cells infiltrated, and inflammatory factors significantly increased, which means it proved that the liver tissues after IRI did have structural changes. But pemafibrate can alleviate inflammation and protect the liver to a certain extent.

The release of ROS could stimulate autophagy and apoptosis in the liver [9, 10]. We determined Beclin-1 and LC3 as well as P62 (autophagy markers) and Bcl-2 family as well as Caspase family (apoptosis markers) by qRT PCR, WB, and immunohistochemistry [13, 28]. We found that PEM injection enabled the injured liver to produce more P62 and Bcl-2 and reduce Beclin-1, LC3, Bax, Caspase 9, and Caspase 3. These results indicated that PEM could alleviate the programmed cell death. Autophagy is a continuous cytological behavior. In the study of the molecular mechanism of autophagy, a large number of autophagy-related proteins have gradually been discovered and given the same name. These ATG proteins play an important role in both the initiation of autophagy and the production of autophagosomes [29, 30]. P62 and LC3 are also key proteins for autophagy. P62 is considered to be an autophagy-specific substrate that can interact with LC3 to enter the autophagosome and is degraded by the lysosome in the autophagosome. Beclin-1 combines with BCL-2 to form a Beclin-1/BCL-2 complex [31, 32]. It can be seen that autophagy and apoptosis are closely related. In the process of apoptosis, mitochondria are considered to be at the center of apoptosis regulation. Bcl-2 and Bax, as important members, participate in regulating, releasing Cyto C, and recruiting Caspase 9 [33, 34]. Caspase 9 belongs to the apoptosis-initiating subclass, but Caspase 3 belongs to the apoptotic effect subclass [35, 36]. In other words, Caspase 9 is located at the upstream of Caspase 3 and can regulate its protein level.

**Figure 6:**
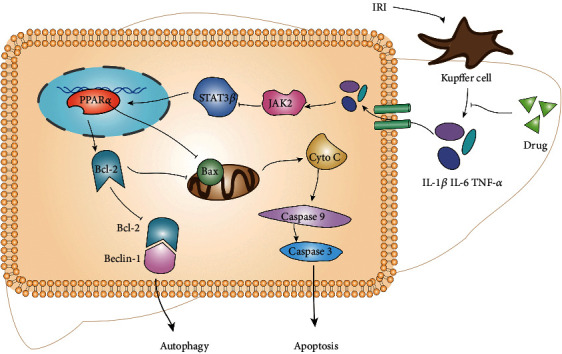
Kupffer cells produce a number of inflammatory mediators, which can bind to cell membrane receptors, promote JAK2, and inhibit p-STAT3*β* and PPAR alpha. Once PPAR*α* was inhibited, Bcl-2 decreased, and Bax increased, which could stimulate apoptosis and autophagy. Pemafibrate can inhibit the release of inflammatory factors and reduce the subsequent reactions. It could protect the liver from excessive programmed cell death by increasing the levels of p-STAT3*β* and PPAR*α* in the hepatic IRI.

Since the expression level of STAT3*α* : STAT4 *β* is 4 : 1, most studies ignore the role of STAT3*β* and regard STAT3*α* as STAT3 study [33, 34]. The two subtypes were determined to further understand the expression of the two subtypes. In our experiment, compared with the IRI group, the levels of JAK2 and p-STAT3*α* decreased, while the p-STAT3*β* remarkably increased, and STAT3 had no change. The significant difference between the two isoforms of p-STAT3 indicated that the two have opposite effects, and p-STAT3*β* has a positive effect on liver damage, which meant that the upstream stimulator acts on JAK2 and then stimulates p-STAT3*β*, not STAT3*β* and STAT3*α*. This might be related to p-STAT3*β*:p-STAT3*α* heterodimer and p-STAT3*α*:p-STAT3*α* homodimer [34]. The decrease of JAK2 resulted in the decrease of p-STAT3*α* and the increase of monomerized p-STAT3*β*. According to previous researches, JAK2/STAT3*β* plays an important role in the immune response. The activation of macrophages caused by IRI injury produces a substantial number of inflammatory mediators, such as IL-1*β*, IL-6, and TNF-*α* [37–39]. These inflammatory factors can act on receptors on the surface of liver cells, activate JAK2 after entering the cells, and phosphorylate STAT3*β* [40] (Figure 6). Multiple previous studies have shown that the activation of PPAR*α* by IL-6 is mediated by the JAK2/STAT3 pathway [41–43].

Pemafibrate is also important in autophagy and apoptosis [44, 45]. The mechanism of pemafibrate is still unclear, but the inhibitory effect on autophagy is not mediated by SIRT [46]. As a PPAR*α* agonist, PEM might activate ATG and TEFEB, both essential parts of the autophagy process, and inhibit autophagy [46–49]. TEFEB is also involved in immune response and inflammatory response [50]. When PPAR*α* is knocked out, the agonist is not able to reverse the inhibition of autophagy or to induce lipid degradation and lipid phagocytosis [51]. PPAR*α* binds to RXR to form a dimer to regulate of the target genes. The inhibited expression of PPAR*α* would lead to the weakened antiapoptotic effect of the drug and the lost of protection of mitochondria [52]. DOX-DNA complex and DOX-TOP2*β* complex can inhibit the expression of PPAR*α*, cause cell apoptosis, and promote the release of ROS. PEM is also closely related to inflammation [53–59]. The myocardial IRI will lower the level of PPAR*α* and increase the secretion of ROS by myocardium [60, 61]. After the expression of PPAR*α* was inhibited, mice are more susceptible to oxidative stress. Our experimental results also showed that PEM can alleviate autophagy and apoptosis, and the key molecules such as Bcl-2, Bax, Caspase 3, Caspase 9, LC3, P62, and Beclin-1 all had corresponding changes. Besides, the level of IL-1*β*, IL-6, and TNF-*α* rose with the increase of PPAR*α*. These results suggested that the effect of PEM was dose-dependent, which indicated that the anti-inflammatory, antiapoptotic, and antiautophagic effects of three doses (0.1 m/kg, 0.5 mg/kg, and 1.0 mg/kg) increase in turn.

In summary, our study found that HIRI stimulates liver Kupffer cells to release amount of inflammatory factors, which enter liver cells by binding to cell membrane receptors. These inflammatory mediators promote the secretion of JAK2 and the phosphorylation of STAT3*α* and inhibit the phosphorylation of STAT3*β*. When pemafibrate intervened, the above reaction was reversed. Increased levels of p-STAT3*β* activate PPAR*α*, thereby inhibiting cell apoptosis and autophagy. There are some limitations in our research. For example, the relationship between pemafibrate and JAK2/STAT3 *β* still needs further study. Whether pemafibrate is safe in clinical treatment is unknown. Its therapeutic effect needs to be compared with the commonly used drugs in clinic.

## 5. Conclusions

Our study found that pemafibrate can effectively inhibit IR damage to the liver of Balb/c mice. In our experiments, pemafibrate significantly reduces serum ALT and AST levels and relieves liver pathophysiological changes. In addition, pemafibrate inhibits the release of inflammatory factors, including IL-1*β*, IL-6, and TNF-*α*, as well as cell deaths such as apoptosis and autophagy by regulating JAK2/STAT3*β*/PPAR*α*. In conclusion, our research suggests that pemafibrate could be a potential therapeutic agent for HIRI.

## Data Availability

The data used to support the findings of this study are available from the corresponding author upon request.
