# Correction to “Pemafibrate Pretreatment Attenuates Apoptosis and Autophagy During Hepatic Ischemia–Reperfusion Injury by Modulating JAK2/STAT3*β*/PPAR*α* Pathway”

**DOI:** 10.1155/ppar/9841376

**Published:** 2026-01-31

Z. Cheng and C. Guo, “Pemafibrate Pretreatment Attenuates Apoptosis and Autophagy During Hepatic Ischemia–Reperfusion Injury by Modulating JAK2/STAT3*β*/PPAR*α* Pathway,” *PPAR Research* 2021 (2021): 6632137, https://doi.org/10.1155/2021/6632137.

1. The IL‐1*β*‐2 h and TNF‐*α*‐8 h images in Figure 3c have been duplicated. Upon checking the original data and experimental protocols, we found that there was no problem with the IL‐1*β* data. However, there was an error when duplicating the TNF‐*α* data. All three images were cropped from the same original image, probably due to mislabeling of the file name, which resulted in the images being pasted incorrectly. As shown below, replace the original image in the red box above with the image in the red box below (named “TNF‐*α*‐8 h” in the “raw data”).





2. There are duplicate JAK2‐2 h and JAK2‐8 h images in Figure 5a. We regret that the same error occurred as above. This was caused by incorrect pasting. As shown below, replace the original image in the red box above with the image in the red box below (named “JAK2‐8 h” in the “raw data”).





**Data Availability Statement**

The data used to support the findings of this study are available from the corresponding author upon request.

We apologize for this error.

